# Multilevel medical insurance mitigate health cost inequality due to air pollution: Evidence from China

**DOI:** 10.1186/s12939-024-02238-9

**Published:** 2024-08-06

**Authors:** Ennan Wang, Minglai Zhu, Yisha Lin, Xiaoyu Xi

**Affiliations:** 1 School of Health Policy and Management, Nanjing Medical University, 101 Longmian Avenue, Nanjing, 211166 Jiangsu China; 2 School of Finance, Nankai University, 38 Tongyan Avenue, Tianjin, 300350 China; 3 The Research Center of National Drug Policy & Ecosystem, China Pharmaceutical University, 639 Longmian Avenue, Nanjing, 211198 Jiangsu China

**Keywords:** Air pollution, Health equity, Multilevel medical insurance systems, Household income

## Abstract

**Background:**

Air pollution affects residents’ health to varying extents according to differences in socioeconomic status. However, there has been a lack of research on whether air pollution contributes to unfair health costs.

**Methods:**

In this research, data from the China Labour Force Dynamics Survey are matched with data on PM2.5 average concentration and precipitation, and the influence of air pollution on the health expenditures of residents is analysed with econometric methods involving a two-part model, instrument variables and moderating effects.

**Results:**

The findings reveal that air pollution significantly impacts Chinese residents’ health costs and leads to low-income people face health inequality. Specifcally, the empirical evidence shows that air pollution has no significant influence on the probability of residents’ health costs (*β* = 0.021, *p* = 0.770) but that it increases the amount of residents’ total outpatient costs (*β* = 0.379, *p* < 0.006), reimbursed outpatient cost (*β* = 0.453, *p* < 0.044) and out-of-pocket outpatient cost (*β* = 0.362, *p* < 0.048). The heterogeneity analysis of income indicates that low-income people face inequality due to health cost inflation caused by air pollution, their total and out-of-pocket outpatient cost significantly increase with PM2.5 (*β* = 0.417, *p* = 0.013; *β* = 0.491, *p* = 0.020). Further analysis reveals that social basic medical insurance does not have a remarkable positive moderating effect on the influence of air pollution on individual health inflation (*β* = 0.021, *p* = 0.292), but supplementary medical insurance for employees could reduce the effect of air pollution on low-income residents’ reimbursed and out-of-pocket outpatient cost (*β*=-1.331, *p* = 0.096; *β*=-2.211, *p* = 0.014).

**Conclusion:**

The study concludes that air pollution increases the amount of Chinese residents’ outpatient cost and has no significant effect on the incidence of outpatient cost. However, air pollution has more significant impact on the low-income residents than the high-income residents, which indicates that air pollution leads to the inequity of medical cost. Additionally, the supplementary medical insurance reduces the inequity of medical cost caused by air pollution for the low-income employees.

## Introduction

Economic growth has not only improved the living standards of residents but also greatly improved their health. However, the environmental pollution that accompanies economic development also threatens residents’ health. Based on the Global Burden of Disease (GBD) research, 6.7 million deaths were attributed to indoor and outdoor pollution worldwide in 2019, and of these, 4.3 million people died prematurely because of outdoor air pollution [1]. Water pollution and air pollution have become important factors of the increase in pathogens and health expenditures [2]. Weng et al. [3] project that if China maintains the same level of air quality as in 2017 from 2020 to 2035, healthcare expenditures in the eastern, central, and western regions of China could save ¥35.37 billion, ¥4.64 billion, and ¥3.11 billion, respectively, by the year 2035.

Research on the relationship between air pollution and health has gradually become abundant in the field of health economics during recent years [4, 5], and the related studies cover the following issues. First, regarding the subjects of these studies, some concentrate on the effect of air pollution on vulnerable healthy people, such as infants, children and elderly people [6–9]. On the other hand, the health influence of air pollution is manifold, and the previous studies emphasise mortality [10–12], lifespan [13] and chronic diseases [14–16]. Some current studies focus on the health problems caused by air pollution, but only a few studies analyse the relationship between air pollution and medical expenditures, especially with Chinese provincial-level data. Zeng & He [17] use a spatial lag model to estimate the impact of industrial air pollution on health care expenditures in Chinese provinces and conclude that the increasing spatial agglomeration of provincial health care expenditures is caused by the joint effect of industrial air pollution, health reform, and other socioeconomic factors. Wu et al. [18] use a dynamic threshold panel model to study a potential nonlinear relationship between environmental pollution and urbanization in the context of different health costs of residents, and they find that environmental pollution has inhibited the improvement of comprehensive urbanization, population urbanization, economic urbanization, and living conditions urbanization but promoted living environment urbanization.

Although air pollution has a negative impact on residents’ health, the extent of this impact varies according to residents’ income and urban-rural differences. The conventional explanation for this phenomenon posits that individuals with lower socioeconomic status are more susceptible to the adverse effects of air pollution compared to their counterparts with higher socioeconomic status. At the macro level, pollution levels may be higher in economically disadvantaged regions. Samoli et al. [19] observed that elevated pollution levels in regions with a higher proportion of individuals born outside EU28, higher crime rates, or increased unemployment rates suggest that poorer air quality is generally experienced in deprived urban areas across Europe. Jorgenson et al. [20] illustrate that the harmful effect of fine particulate matter on life expectancy is especially pronounced in states with both very high levels of income inequality and very large black populations. Consistent with the research results found in other countries, relevant studies in China also indicate that air pollution exacerbates health inequality among residents. Yang et al. [21] find health inequality is prevalent throughout China and the damage to health caused by pollution further increases the levels of health inequality to varying degrees in groups with different income levels. More specifically, Liu et al. [22] confirm the existence of the health channel through which air pollution exerts influence on income inequality. Xie et al. [23] compare the impact of PM2.5 on China’s economy and the health of the Chinese people, and find that in the underdeveloped areas of the western economy, the health problems caused by environmental pollution are more serious than those in the more developed eastern provinces; this phenomenon became more obvious after the implementation of environmental regulations.

However, some studies present the opposite view, namely, that people in economically developed areas may be exposed to more polluted environments than others for a longer time and thus be affected by more air pollution [24, 25]. Furthermore, the difference between urban and rural areas is one of the factors influencing the degree to which residents’ health is impacted by air pollution. For example, Yang & Liu [21] find that the health inequalities caused by pollution may be exacerbated in rural areas.

Moreover, some researchers also discuss the impact of air pollution on commercial health insurance and find that there is a causal relationship between air pollution and medical insurance demand: the more serious air pollution there is, the more commercial health insurance demand there is [26, 27]. However, so far, there is a lack of research on the role that insurance plays in the impact of air pollution on the health and health costs of residents, whether social medical insurance or commercial health insurance.

This paper focuses on how air pollution affects residents’ health costs and how residents’ income levels and health care play a role in this process. To realize our research objective, we use a two-part model and instrumental variables to confirm the presence of a causal relationship between air pollution and health costs, and we explore the existence of unfair medical costs and the role of multilevel medical insurance with subgroup regressions and by examining moderating effects.

This paper makes the following contributions. First, while previous studies have predominantly focused on the relationship between air pollution and health [10, 12, 14, 16, 20], this paper extends the impact of air pollution to medical expenditures. Unlike prior studies investigating the connection between pollution and health expenditures in China, which relied on macro-level provincial data [5, 23], our approach involves the integration of urban pollution data with micro-level dynamic survey data of Chinese laborers. This enables us to leverage pollution data from more than 300 cities in China, thereby augmenting the richness and comprehensiveness of our results. Second, while previous research has primarily analyzed whether air pollution leads to health inequality among residents [19–23], this paper, under the premise of controlling for health conditions, places emphasis on investigating whether health inequality further translates into inequality in health expenditures. Finally, the most pivotal innovation in this paper lies in examining whether different types of medical insurance can ameliorate the inequality faced by residents due to air pollution. Prior studies have largely overlooked the role of health insurance in moderating disparities in medical expenditures [28]. Leveraging the characteristics of China’s multilevel medical insurance system, this paper sequentially analyzes whether social basic medical insurance and supplementary medical insurance can mitigate inequality in residents’ health expenditures caused by air pollution.

This paper is organized as follows. Section 2 describes the empirical strategy of this study and presents the research data. Section 3 presents the results of the empirical analysis of the effect of air pollution on medical expenditures and robustness tests of the basic results. Section 4 discusses the results and further empirical research. Section 5 presents the conclusion.

## Methodology

### Empirical strategy

According to previous studies, many of the diseases caused by air pollution are cardiovascular, respiratory and psychological in nature [14, 16], and most of them are chronic diseases and treated in outpatient clinics. To explore the effect of air pollution on the medical burdens of residents, this paper takes the total outpatient expenses, reimbursements and out-of-pocket expenses of residents over the past two weeks as explanatory variables.

China promulgated the *Ambient Air Quality Standards* (GB3095-2012) in February 2012, increased the number of pilot cities adopting the 2012 edition of the new standards from 74 in 2012 to 161 in 2014, and implemented the standards nationwide in January 2016. Because most of these pilot cities are provincial capitals or developed cities, if we use the air pollution index (Air Quality Index, AQI) published on the China Environmental Monitoring website, we will have biased samples and results. On the other hand, particulate matter is the most important air pollutant in China, as particles smaller than 2.5 μm relatively easily enter indoor spaces and harm residents’ health [23, 29]. Therefore, we select the mean PM_2.5_ concentration in the examined cities as the core explanatory variable of our econometric model.

If the residents are not ill during the sample period, many observations of the explained variables, that is, the random discrete-continuous variables (the discrete part is zero, the continuous part is medical expenditures), will be equal to zero, which violates the assumption that the error term needs to satisfy the normal distribution. Thus, we adopt a two-part model (TPM), which is widely used in health economics research [30, 31] when medical expenditures are used as an explained variable. The first part of the TPM is the probit model shown in Eq. (1), in which the explained variable is the probability of occurrence and where $\:{x}$,$\:\:{\delta\:}$ and F are the explanatory variable, the estimated parameter vector and the cumulative distribution function, respectively.

1 $$\:\begin{array}{*{20}{c}}{\phi \:(y > 0) = {\rm{Pr}}\left( {y > 0|x} \right) = F\left( {x\delta } \right)}\end{array}$$

The linear regression model when y is positive in the second part of the TPM is shown in Eq. (2). In Eq. (2), $\:{x}$ and $\:{\gamma\:}$ also represent vectors of the explanatory variables and estimated parameters, respectively, and $\:g$ is the density function at $\:y|y>0$.

2 $$\:\begin{array}{*{20}{c}}{\phi \:\left( {y|y > 0,x} \right) = g\left( {x\gamma } \right)}\end{array}$$

Then, the likelihood distribution of each variable can be written as Eq. (3) and further as formula (4), where $i{\rm{(}} \cdot {\rm{)}}$ is an indicated function.

3 $$\:\phi \:\left( y \right) = {\left\{ {1 - F\left( {x\delta } \right)} \right\}^{i\left( {i = 0} \right)}} \times \:{\left\{ {F\left( {x\delta } \right)g\left( {x\gamma } \right)} \right\}^{i\left( {y > 0} \right)}}$$

4 $$\:\begin{array}{*{20}{c}}\begin{array}{l}{\rm{ln}}\left\{ {\phi \:\left( y \right)} \right\} = i\left( {i = 0} \right){\rm{ln}}\left\{ {1 - F\left( {x\delta } \right)} \right\}\\+ i\left( {i > 0} \right)\left[ {{\rm{ln}}\left\{ {F\left( {x\delta } \right)} \right\} + {\rm{ln}}\left\{ {g\left( {x\gamma \:} \right)} \right\}} \right]\end{array}\end{array}$$

In our study, the probability model of the first part of the TPM is shown in Eq. (5). In Eq. (5), $\:y$ is total, reimbursed and out-of-pocket medical expenditures, and $\:i$ represents the observation samples.$\:\:{PM}_{ct}$ is the concentration of PM_2.5_ in each individual’s city, and $\:{X}_{it}^{{\prime\:}}$ is a control variable that includes not only demographic variables such as age, gender, marital status, and education level but also health-related variables such as self-rated health, illnesses, smoking, drinking, and exercise habits. Moreover, the key explained variable is PM_2.5_, which is related to the economic development of each city, and we add the per capita GDP of each city. Similarly, the explained variables are outpatient medical expenditures that are influenced by local medical resources and medical insurance, and we control for medical insurance using the number of individuals enrolled and the number of medical institutions per capita of the city. $\:{G}{X}{{\prime\:}}$ represents the per capita GDP and per capita medical institutions of the city.

5 $$\:\begin{array}{*{20}{c}}{{\rm{Pr}}\left( {y > 0|{X_{it}}} \right) = F\left( {\delta \:P{M_{ct}} + \theta \:X_{it}^{\prime \:} + \rho \:G{X^{\prime \:}}_{c,t} + + \mu {\:_t} + \epsilon {\:_{it}}} \right)}\end{array}$$

We choose ordinary least squares (OLS) in the second part of TPM to estimate medical expenditures, as shown in Eq. (6). The core explanatory variable and control variables are consistent with those of the selection model. Both the selection model and the expenditure model are clustered at the city level.

6 $$\:\begin{array}{*{20}{c}}{E\left( {y|y > 0,x} \right) = {g^{ - 1}}\left( {\alpha \:P{M_c} + \beta \:X_{it}^{\prime \:} + \gamma \:G{X^{\prime \:}}_{c,t} + \mu {\:_t} + {\epsilon _{ict}}} \right)}\end{array}$$

To ensure the integrity and richness of the research data, a mixed regression (pooled regression) model is proposed. Therefore, before the formal econometric analysis, it is necessary to ensure that there are no fixed effects. When a fixed-effect regression is used, the results of the F-test show that the original hypothesis “$\:{H}_{0}:all\:{u}_{i}=0$” is accepted and that the mixed regression is appropriate.

### Data and variables

The data are composed of the following two parts. The micro data are from the 2014–2016 China Labour Force Dynamics Survey (CLDS) conducted by the Social Sciences Survey Center of Sun Yat-sen University. There are two reasons for choosing the CLDS data. First, the CLDS focuses on the current situation of and changes in China’s labour force. In addition to containing basic statistical indicators such as those on education, work, population mobility and health, it contains relatively detailed medical expenditure data and medical insurance data that meet our research needs. Second, compared with other open micro-databases, CLDS is more accessible in terms of using city codes rather than provincial codes. Thus, we can match urban environmental data such PM_2.5_ and rainfall relatively accurately, which improves the accuracy of our research results.

The second part is macro data regarding PM_2.5_ and rainfall. The data on concentrations of PM_2.5_ come from the Center for International Earth Science Information Network (CIESIN) hosted by Columbia University. The PM_2.5_ published by CIESIN covers more sample cities than the AQI published by the China National Environmental Monitoring Center and avoids artificially selecting samples. Furthermore, we choose rainfall as the instrumental variable of PM_2.5_. The rainfall data mainly come from the statistical yearbooks of various provinces and cities, and the few missing data in these statistical yearbooks are filled in with data from the Water Resources Bulletin (WRB) published by the water resources departments of the provinces and cities.

All the PM_2.5_ and rainfall data are matched with the CDLS data using city codes. After deleting the missing data and outliers, the sample contains 39,366 observations. In addition, the self-reported health status score is 1 when the examined residents’ health state is excellent, 2 is very good, 3 is good, 4 is bad and 5 represents very bad in the original CLDS questionnaire. To simplify its explanation, the self-rating health status score used in our research is equal to six minus the original self-rating health status score in the CLDS data; thus, each individual’s health status is transformed to make it proportional to the corresponding self-assessment score. The sample statistics are described in Table 1.

**Table 1:** Summary statistics

Variable	Definition	Mean	Std. Dev.	Min	Max
gender	Gender	0.480	0.500	0	1
marriage	Marital status	0.840	0.370	0	1
age	Age	44.56	14.51	5	114
age2	Square of age	2 196	1 265	25	12 996
edu	Highest education	3.580	2.290	1	11
ur	Urban or rural	0.630	0.480	0	1
fn	Number of family members	4.530	1.980	1	20
hs	Self-rated health status	3.680	0.980	1	5
pain	Number of pains in a month	1.940	1.110	1	5
drink	Weekly drinking (Yes/No)	0.190	0.400	0	1
smoke	Weekly smoking (Yes/No)	0.270	0.440	0	1
exercise	Weekly exercise (Yes/No)	0.270	0.440	0	1
sbmi	Type of social basic medical insurance	0.190	0.690	0	4
omi	Other medical insurance purchased	2.240	1.210	0	4
inc	Per capita family income	6.850	0.720	4.191	7.942
GDP	Per capita GDP	10.78	0.720	9.080	13.06
med_ins	Number of medical institutions per capita	5.360	0.720	3.760	7.510
PM	Concentration of PM_2.5_	3.550	0.450	2.330	4.400
rainfall	Rainfall	6.880	0.640	5.340	7.900
Pre_PM	PM_2.5_ during previous year	3.600	0.480	2.200	4.470
Pre_rainfall	Rainfall during previous year	6.850	0.640	5.050	7.940
med_exp	Outpatient medical expenditures during the past two weeks	0.310	1.390	0	11.78
clm_exp	Outpatient claim expenditures during the past two weeks	0.210	1.130	0	11.70
oop_exp	Outpatient out-of-pocket medical costs during the past two weeks	0.180	1.050	0	11.16

Notes: inc, GDP, med_ins, PM, rainfall, pre_PM, pre_rainfall, med_exp, clm_exp and oop_exp are added to one and the logarithm is taken

In addition to analysing the impact of air pollution on residents’ medical expenditures, we explore whether air pollution makes residents with different incomes face unfair health costs and how medical insurance affects these costs. Therefore, median household per capita income was used as a grouping variable to compare the key variables of the residents in high- and low-income groups, as shown in Table 2.

**Table 2:** Descriptive statistics of the key variables of different income groups

variable	Low-income group	mean	High-income group	mean	Differences
sbmi	21,481	2.483	17,885	1.951	0.531^***^
omi	21,481	0.0900	17,885	0.320	-0.229^***^
inc	21,481	8.025	17,885	10.05	-2.022^***^
GDP	21,481	10.57	17,885	11.04	-0.473^***^
med_ins	21,481	5.363	17,885	5.349	0.014^*^
PM	21,481	3.531	17,885	3.564	-0.033^***^
rainfall	21,481	6.832	17,885	6.946	-0.113^***^
Pre-PM_2.5_	21,481	3.584	17,885	3.613	-0.029^***^
Pre-rainfall	21,481	6.811	17,885	6.888	-0.077^***^
med_exp	21,481	0.365	17,885	0.253	0.112^***^
clm_exp	21,481	0.240	17,885	0.163	0.078^***^
oop_exp	21,481	0.210	17,885	0.150	0.061^***^

*Notes: The t-statistics in parentheses are as follows*: ^*^significant at the 10% level, ^**^significant at the 5% level, and^***^significant at the 1% level

From the perspective of medical insurance enrolment, the proportion of low-income residents covered by social basic medical insurance with lower financing and treatment levels, such as Urban Resident Basic Medical Insurance (URBMI) and the New Rural Cooperative Medical System (NRCMS), is higher than that of high-income residents covered by these plans, while the proportion of low-income residents enrolled in supplementary medical insurance and commercial health insurance is lower. As might be expected, the average PM2.5 concentrations experienced by the low-income group is lower than those facing the high-income group; however, the total outpatient expenses, reimbursements and out-of-pocket expenditures of the low-income group are significantly higher than those of the high-income group.

## Results

### Basic results

Table 3 presents the basic results for the whole sample. We use the total, claimed and out-of-pocket outpatient expenditures during the past two weeks as explained variables and the PM_2.5_ of the sampled cities as explanatory variables. The results reported in Table 3 suggest that the incidence and amount of medical expenditures increase with PM_2.5_. According to the probit estimates, the results are not significant (columns 1–3). In the OLS estimates, medical expenditures, reimbursements, and out-of-pocket costs increase by 0.379%, 0.453% and 0.362%, respectively, with a 1% increase in the PM_2.5_ concentration (column 4–6).

**Table 3:** Basic results: effect of PM_2.5_ on medical expenditures

	Probit			OLS		
	(1)	(2)	(3)	(4)	(5)	(6)
PM_2.5_	0.021	0.070	0.006	0.379^***^	0.453^**^	0.362^**^
	(0.29)	(0.83)	(0.07)	(2.73)	(2.01)	(1.98)
age	-0.000	0.002	0.003	0.046^**^	0.048^*^	0.049^**^
	(-0.01)	(0.25)	(0.42)	(2.28)	(1.84)	(2.09)
age2	-0.000	-0.000	-0.000	-0.000^*^	-0.000	-0.000^*^
	(-0.96)	(-0.84)	(-1.19)	(-1.87)	(-1.60)	(-1.83)
gender	0.006	-0.025	0.054	0.317^***^	0.463^***^	0.182
	(0.19)	(-0.75)	(1.47)	(3.27)	(3.39)	(1.63)
marriage	-0.060	-0.083	-0.078	-0.012	-0.136	0.151
	(-1.16)	(-1.49)	(-1.34)	(-0.08)	(-0.78)	(0.84)
edu	-0.006	-0.000	-0.013	0.008	-0.005	0.026
	(-0.67)	(-0.00)	(-1.40)	(0.29)	(-0.13)	(0.81)
fn	0.017^***^	0.019^***^	0.020^***^	0.012	0.024	-0.018
	(3.02)	(2.65)	(2.79)	(0.61)	(0.91)	(-0.66)
ur	-0.000	0.015	0.029	-0.383^***^	-0.387^***^	-0.466^***^
	(-0.00)	(0.27)	(0.60)	(-3.83)	(-2.72)	(-3.69)
hs	-0.279^***^	-0.281^***^	-0.246^***^	-0.207^***^	-0.152^**^	-0.192^***^
	(-18.04)	(-15.71)	(-12.19)	(-4.33)	(-2.35)	(-3.74)
pain	0.311^***^	0.317^***^	0.274^***^	0.187^***^	0.200^***^	0.142^***^
	(19.81)	(17.53)	(14.16)	(4.65)	(3.69)	(3.11)
smoke	-0.067^*^	-0.014	-0.086^**^	-0.099	-0.274^*^	-0.037
	(-1.86)	(-0.32)	(-2.03)	(-0.79)	(-1.69)	(-0.24)
drink	-0.107^**^	-0.114^**^	-0.066	0.074	0.030	0.047
	(-2.53)	(-2.40)	(-1.45)	(0.62)	(0.20)	(0.29)
exercise	0.056^**^	0.090^***^	0.060^*^	0.109	0.064	-0.059
	(2.00)	(2.90)	(1.72)	(1.19)	(0.55)	(-0.47)
inc	0.004	-0.004	0.011	0.040	0.061^*^	0.045
	(0.50)	(-0.42)	(1.24)	(1.44)	(1.85)	(1.17)
GDP	-0.059^**^	-0.004	-0.013	0.216^***^	0.004	-0.029
	(-1.99)	(-0.09)	(-0.39)	(3.36)	(0.03)	(-0.35)
med_ins	0.003	0.027	-0.031	-0.008	-0.102	0.014
	(0.11)	(0.72)	(-0.92)	(-0.15)	(-1.35)	(0.24)
cons	-0.902^*^	-2.401^***^	-1.268^**^	0.856	2.981^*^	2.963^**^
	(-1.80)	(-3.60)	(-2.28)	(0.83)	(1.92)	(2.48)
sbmi	Yes	Yes	Yes	Yes	Yes	Yes
omi	Yes	Yes	Yes	Yes	Yes	Yes
Year effect	Yes	Yes	Yes	Yes	Yes	Yes
District fixed-effect	Yes	Yes	Yes	Yes	Yes	Yes
No. of observations	39,366	39,366	39,366	2094	1388	1275
R^2^	0.185	0.221	0.177	0.187	0.165	0.203

Notes: (1) Columns (1)-(3) present the incidence of total, reimbursed and out-of-pocket outpatient medical expenditures. Columns (4)-(6) present the amounts of total, reimbursed and out-of-pocket outpatient medical expenditures. (2) The figures in parentheses are clustered robust t-statistics at the level of cities. (3) The t-statistics in parentheses are as follows: ^*^ significant at the 10% level, ^**^significant at the 5% level, and ^***^significant at the 1% level

### Robustness tests

#### Hysteresis test

The analysis so far has been based on the mean PM_2.5_ concentrations during each year. However, some research suggests that the effect of PM_2.5_ on health takes some time, and its influence on medical expenditures may also be lagged. Thus, we use the mean PM_2.5_ concentrations in 2013 and 2015 as the core explanatory variables (the original values used are from 2014 to 2016), and the results are shown in Table 4. The results suggest that the PM_2.5_ the year before each examined year also has a significant effect on the amount of outpatient expenses in that year. More specifically, the total, reimbursed and out-of-pocket outpatient medical expenditures increase by 0.337%, 0.52% and 0.318% with a 1% increase in PM_2.5_, respectively. When compared with Table 3, it can be observed that the coefficients of total and out-of-pocket medical expenditures are consistent with those of the basic results, and only the coefficient of reimbursed expenses increases slightly. The hysteresis test suggests that the effect of PM_2.5_ on medical costs is robust.

**Table 4:** Robustness test: effect of PM_2.5_ during the previous year on medical expenditures

	Probit			OLS		
	(1)	(2)	(3)	(4)	(5)	(6)
Pre_PM_2.5_	-0.050	-0.013	-0.034	0.377^***^	0.520^***^	0.318^*^
	(-0.73)	(-0.15)	(-0.42)	(2.95)	(2.71)	(1.79)
age	-0.000	0.002	0.003	0.046^**^	0.049^*^	0.049^**^
	(-0.06)	(0.21)	(0.40)	(2.29)	(1.87)	(2.12)
age2	-0.000	-0.000	-0.000	-0.000^*^	-0.000	-0.000^*^
	(-0.91)	(-0.80)	(-1.16)	(-1.89)	(-1.64)	(-1.85)
gender	0.005	-0.027	0.053	0.318^***^	0.462^***^	0.182
	(0.16)	(-0.78)	(1.47)	(3.28)	(3.38)	(1.62)
marriage	-0.058	-0.081	-0.078	-0.009	-0.133	0.153
	(-1.13)	(-1.46)	(-1.33)	(-0.06)	(-0.76)	(0.85)
edu	-0.006	0.000	-0.013	0.009	-0.005	0.026
	(-0.64)	(0.03)	(-1.39)	(0.30)	(-0.13)	(0.81)
fn	0.018^***^	0.019^***^	0.020^***^	0.012	0.024	-0.018
	(3.03)	(2.67)	(2.80)	(0.60)	(0.91)	(-0.64)
ur	0.003	0.018	0.031	-0.383^***^	-0.392^***^	-0.462^***^
	(0.06)	(0.32)	(0.63)	(-3.82)	(-2.76)	(-3.70)
hs	-0.280^***^	-0.282^***^	-0.247^***^	-0.207^***^	-0.151^**^	-0.193^***^
	(-18.05)	(-15.69)	(-12.19)	(-4.33)	(-2.32)	(-3.77)
pain	0.311^***^	0.316^***^	0.274^***^	0.186^***^	0.201^***^	0.140^***^
	(19.75)	(17.48)	(14.14)	(4.64)	(3.70)	(3.10)
smoke	-0.066^*^	-0.014	-0.086^**^	-0.102	-0.274^*^	-0.039
	(-1.84)	(-0.31)	(-2.03)	(-0.81)	(-1.69)	(-0.25)
drink	-0.107^**^	-0.114^**^	-0.066	0.075	0.030	0.048
	(-2.53)	(-2.39)	(-1.46)	(0.63)	(0.20)	(0.30)
exercise	0.057^**^	0.092^***^	0.061^*^	0.111	0.070	-0.054
	(2.04)	(2.95)	(1.74)	(1.21)	(0.59)	(-0.43)
inc	0.004	-0.004	0.011	0.039	0.060^*^	0.043
	(0.50)	(-0.42)	(1.24)	(1.41)	(1.83)	(1.14)
GDP	-0.056^*^	0.000	-0.012	0.220^***^	-0.003	-0.023
	(-1.89)	(0.01)	(-0.37)	(3.41)	(-0.03)	(-0.29)
med_ins	0.007	0.030	-0.030	0.004	-0.099	0.030
	(0.23)	(0.80)	(-0.87)	(0.08)	(-1.34)	(0.56)
cons	-0.667	-2.135^***^	-1.132^**^	0.741	2.760^*^	2.955^**^
	(-1.33)	(-3.17)	(-1.98)	(0.69)	(1.75)	(2.34)
sbmi	Yes	Yes	Yes	Yes	Yes	Yes
omi	Yes	Yes	Yes	Yes	Yes	Yes
Year effect	Yes	Yes	Yes	Yes	Yes	Yes
District fixed-effect	Yes	Yes	Yes	Yes	Yes	Yes
No. of observations	39,366	39,366	39,366	2094	1388	1275
R^2^	0.185	0.221	0.177	0.187	0.166	0.202

Notes: (1) Columns (1)-(3) present the incidence of total, reimbursed and out-of-pocket outpatient medical expenditures. Columns (4)-(6) present the amounts of total, reimbursed and out-of-pocket outpatient medical expenditures. (2) The figures in parentheses are clustered robust t-statistics at the level of cities. (3) The t-statistics in parentheses are as follows: ^*^ significant at the 10% level, ^**^ significant at the 5% level, and ^***^ significant at the 1% level

#### Endogenous test

As we mention in the introduction, the degree of air pollution in a city is closely related to its economic development. The economic development of an area not only improves the health status of its residents, such as their life expectancy and health level, through an improved medical security system but also provides economic support for residents’ medical expenditures. Therefore, to avoid negative impacts from missing variables and the endogeneity of PM_2.5_, we choose to employ rainfall as an instrumental variable. First, rainfall is strongly correlated with airborne PM_2.5_: PM_2.5_ concentrations decrease as rainfall increases. After the test, the F-statistics in the first stage of the two-stage least squares (2SLS) model are 46.67, 12.97, and 98.99. Importantly, all of them are greater than 10, meeting the requirements of strong instrumental variables. On the other hand, according to our basic empirical strategy, rainfall is related to the PM_2.5_ concentration in the air but has no direct effect on the health status and medical expenditures of an area’s residents, which suggests that rainfall satisfies exogeneity. Above all, rainfall not only satisfies the correlation with PM_2.5_ but also is exogeneous because it has nothing to do with health or medical expenditures. Therefore, it meets the basic requirements of an instrumental variable.

Since we use probit and OLS models in the TPM, we build IV-Probit and 2SLS models when we employ rainfall as an instrumental variable to test the robustness of the basic results. However, the premise of using instrumental variables is that there is endogeneity in a basic regression. The results of a Durbin-Wu-Hausman (DWH) test suggest that whether a probit or OLS model is used, when we choose PM_2.5_ as core explanatory variable to explore its effect on medical cost, we should accept the original hypothesis that all variables are exogenous; thus, the basic regression results are not extensively affected by this endogeneity.

#### Placebo test

**Fig. 1:**
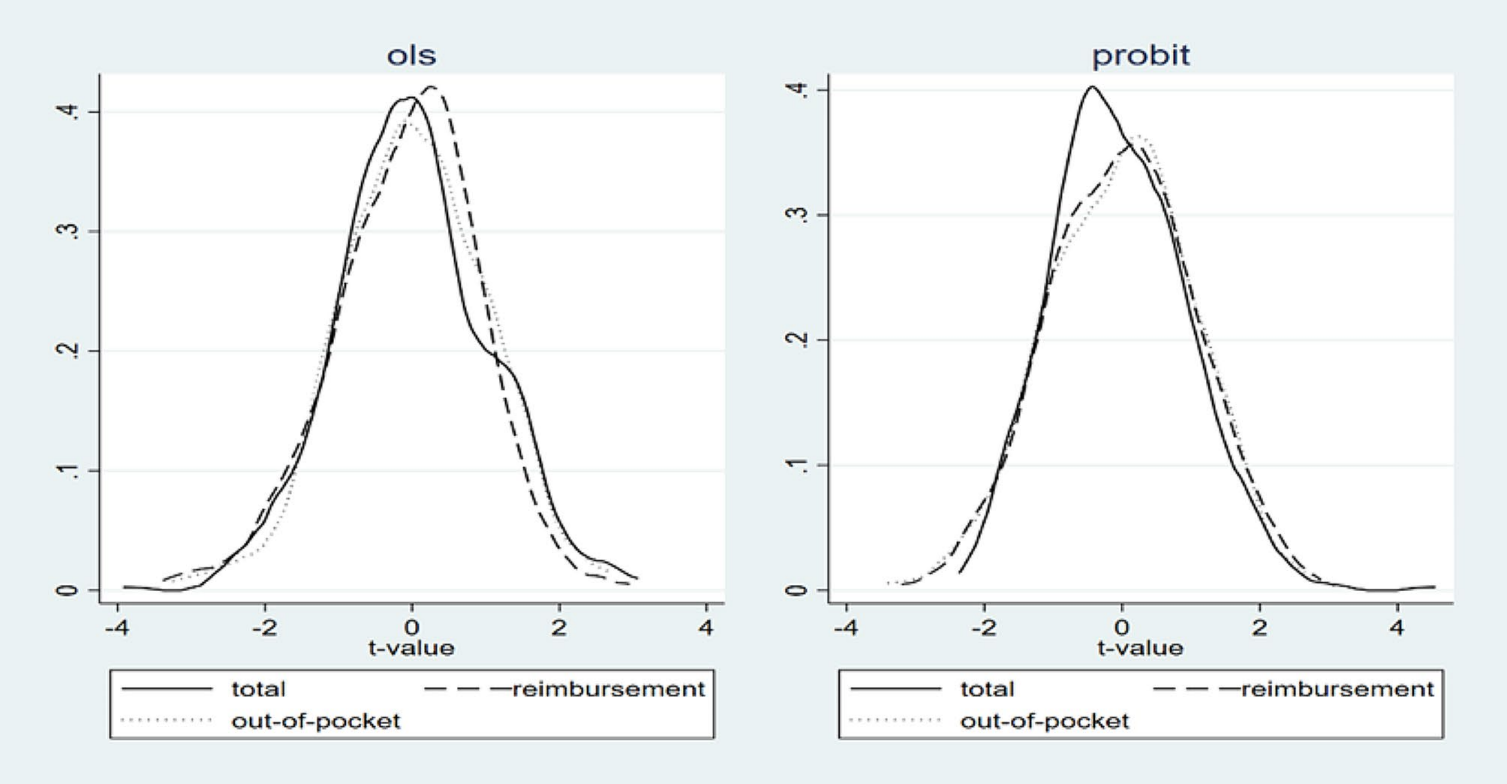
The results of the placebo test

Furthermore, to prove the effect of PM_2.5_ on medical expenditures again, we use a random distribution of the PM_2.5_ concentrations in the examined 150 cities as a placebo to replace the original PM_2.5_ concentration. If using this random distribution of the cities’ PM2.5 concentrations has no significant effect on the examined outpatient medical expenditures, the basic results are proven to be robust. From the results of 500 placebo tests (Fig. 1) where the mean concentration of PM2.5 in each city is distributed randomly, the effects on the incidence and amount of outpatient expenditures are roughly normally distributed, and the t-values are mainly concentrated in the [-1.5, 1.5] interval. The results suggest that the random distribution of PM_2.5_ has no significant effect on medical expenditures and that the basic results are robust.

### Inequality of medical cost

We explore whether differences in income cause inequality in medical costs. To do this, we divide the whole sample into two subsamples based on median income and explore the influence of PM_2.5_ on the medical expenditures of low- and high-income individuals. Tables 5 and 6 present the results for the different subsamples.

The results show that PM_2.5_ has no significant effects on the medical costs of high-income residents, while it significantly affects the total and out-of-pocket medical expenditures of low-income residents. The total and out-of-pocket outpatient expenditures for the low-income group exhibit a respective increase of 0.417% and 0.491% in response to a 1% rise in PM2.5 concentration. These increments surpass those observed in the overall group, which stand at 0.379% and 0.362%, respectively.

**Table 5:** The effect of PM_2.5_ concentration on high-income residents

	Probit			OLS		
	(1)	(2)	(3)	(4)	(5)	(6)
PM_2.5_	-0.106	0.031	-0.083	0.364	0.393	-0.060
	(-1.18)	(0.28)	(-0.74)	(1.25)	(0.85)	(-0.19)
age	-0.002	-0.002	0.003	-0.003	-0.007	0.010
	(-0.14)	(-0.17)	(0.23)	(-0.08)	(-0.14)	(0.23)
age2	-0.000	-0.000	-0.000	0.000	0.000	0.000
	(-0.44)	(-0.28)	(-0.52)	(0.61)	(0.62)	(0.08)
gender	-0.028	-0.078	0.008	0.247^*^	0.306	0.318
	(-0.53)	(-1.35)	(0.13)	(1.91)	(1.54)	(1.62)
marriage	-0.046	-0.032	-0.105	0.328	0.374	0.494^*^
	(-0.53)	(-0.33)	(-1.12)	(1.31)	(1.15)	(1.79)
edu	-0.005	-0.002	-0.002	0.010	0.021	0.028
	(-0.42)	(-0.16)	(-0.19)	(0.29)	(0.44)	(0.71)
fn	0.022^*^	0.021	0.030^**^	-0.035	-0.054	-0.112^**^
	(1.83)	(1.46)	(2.10)	(-0.86)	(-1.12)	(-2.56)
ur	0.012	0.089	0.008	-0.457^***^	-0.346^*^	-0.585^***^
	(0.21)	(1.28)	(0.13)	(-3.19)	(-1.72)	(-3.27)
hs	-0.300^***^	-0.303^***^	-0.276^***^	-0.116	-0.088	-0.030
	(-11.73)	(-9.75)	(-8.38)	(-1.52)	(-0.89)	(-0.31)
pain	0.331^***^	0.328^***^	0.279^***^	0.233^***^	0.233^***^	0.215^***^
	(13.65)	(12.25)	(10.12)	(3.50)	(2.92)	(2.78)
smoke	-0.039	0.007	-0.034	0.044	-0.028	-0.013
	(-0.61)	(0.10)	(-0.43)	(0.21)	(-0.10)	(-0.05)
drink	-0.014	-0.074	0.026	-0.007	0.079	-0.153
	(-0.22)	(-1.01)	(0.39)	(-0.04)	(0.32)	(-0.59)
exercise	0.059	0.098^**^	0.035	0.125	0.226	-0.099
	(1.58)	(2.09)	(0.76)	(0.89)	(1.15)	(-0.51)
inc	0.011	0.027	-0.037	-0.015	0.111	-0.143
	(0.35)	(0.64)	(-0.94)	(-0.10)	(0.55)	(-0.66)
GDP	-0.084^**^	-0.084^*^	-0.036	0.247^**^	0.212	-0.006
	(-2.28)	(-1.91)	(-0.84)	(2.21)	(1.13)	(-0.04)
med_ins	0.038	0.098^**^	-0.001	0.048	-0.019	0.054
	(0.99)	(2.41)	(-0.02)	(0.45)	(-0.13)	(0.44)
cons	-0.418	-2.010^**^	-0.466	0.867	-0.276	5.971^**^
	(-0.56)	(-2.40)	(-0.56)	(0.39)	(-0.10)	(2.35)
sbmi	Yes	Yes	Yes	Yes	Yes	Yes
omi	Yes	Yes	Yes	Yes	Yes	Yes
Year effect	Yes	Yes	Yes	Yes	Yes	Yes
District fixed-effect	Yes	Yes	Yes	Yes	Yes	Yes
No. of observations	17,885	17,885	17,729	752	492	459
R^2^	0.192	0.230	0.177	0.213	0.222	0.230

Notes: (1) Columns (1)-(3) present the incidence of total, reimbursed and out-of-pocket outpatient medical expenditures. Columns (4)-(6) present the amounts of total, reimbursed and out-of-pocket outpatient medical expenditures. (2) The figures in parentheses are clustered robust t-statistics at the level of cities. (3) The t-statistics in parentheses are as follows: ^*^ significant at the 10% level, ^**^ significant at the 5% level, and ^***^ significant at the 1% level

**Table 6:** The effect of PM_2.5_ concentration on low-income residents

	Probit			OLS		
	(1)	(2)	(3)	(4)	(5)	(6)
PM_2.5_	0.101	0.112	0.076	0.417^**^	0.547	0.491^**^
	(1.21)	(1.16)	(0.74)	(2.49)	(1.61)	(2.33)
age	0.002	0.007	0.002	0.059^**^	0.048	0.056^**^
	(0.31)	(0.92)	(0.24)	(2.40)	(1.44)	(2.00)
age2	-0.000	-0.000	-0.000	-0.001^**^	-0.001	-0.001^**^
	(-1.17)	(-1.32)	(-0.95)	(-2.35)	(-1.53)	(-1.98)
gender	0.036	0.010	0.091^**^	0.292^**^	0.514^***^	0.032
	(0.95)	(0.23)	(1.98)	(2.15)	(2.72)	(0.22)
marriage	-0.069	-0.114	-0.062	-0.153	-0.348	-0.049
	(-1.00)	(-1.48)	(-0.76)	(-0.81)	(-1.63)	(-0.20)
edu	-0.010	0.006	-0.025	0.029	-0.023	0.031
	(-0.73)	(0.39)	(-1.59)	(0.46)	(-0.28)	(0.47)
fn	0.015^**^	0.019^**^	0.014^*^	0.029	0.054	0.011
	(2.15)	(2.10)	(1.67)	(1.28)	(1.57)	(0.33)
ur	0.002	-0.039	0.050	-0.191	-0.367^*^	-0.246
	(0.03)	(-0.52)	(0.76)	(-1.29)	(-1.94)	(-1.31)
hs	-0.268^***^	-0.270^***^	-0.227^***^	-0.279^***^	-0.223^***^	-0.308^***^
	(-13.11)	(-11.23)	(-9.19)	(-4.72)	(-2.68)	(-4.40)
pain	0.300^***^	0.311^***^	0.272^***^	0.158^***^	0.162^**^	0.102^*^
	(15.00)	(14.50)	(11.19)	(3.22)	(2.33)	(1.92)
smoke	-0.084^*^	-0.028	-0.116^**^	-0.170	-0.429^**^	-0.014
	(-1.67)	(-0.45)	(-2.18)	(-1.15)	(-2.22)	(-0.07)
drink	-0.182^***^	-0.154^***^	-0.139^**^	0.190	0.082	0.251
	(-3.63)	(-2.70)	(-2.46)	(1.16)	(0.41)	(1.18)
exercise	0.055	0.082^*^	0.091^*^	0.101	-0.016	0.001
	(1.38)	(1.87)	(1.82)	(0.72)	(-0.10)	(0.00)
inc	0.003	-0.005	0.018	0.015	0.020	0.006
	(0.35)	(-0.46)	(1.58)	(0.50)	(0.60)	(0.14)
GDP	-0.038	0.041	0.022	0.103	-0.294^*^	-0.088
	(-1.01)	(0.76)	(0.56)	(1.21)	(-1.95)	(-0.89)
med_ins	-0.006	0.003	-0.031	-0.078	-0.204^**^	-0.052
	(-0.20)	(0.07)	(-0.89)	(-1.13)	(-2.00)	(-0.79)
cons	-1.059^*^	-2.631^***^	-1.636^**^	3.697^***^	8.598^***^	4.299^**^
	(-1.65)	(-2.81)	(-2.48)	(2.69)	(3.77)	(2.50)
sbmi	Yes	Yes	Yes	Yes	Yes	Yes
omi	Yes	Yes	Yes	Yes	Yes	Yes
Year effect	Yes	Yes	Yes	Yes	Yes	Yes
District fixed-effect	Yes	Yes	Yes	Yes	Yes	Yes
No. of observations	21,454	21,454	21,256	1342	896	816
R^2^	0.179	0.217	0.178	0.205	0.206	0.238

Notes: (1) Columns (1)-(3) present the incidence of total, reimbursed and out-of-pocket outpatient medical expenditures. Columns (4)-(6) present the amounts of total, reimbursed and out-of-pocket outpatient medical expenditures. (2) The figures in parentheses are clustered robust t-statistics at the level of cities. (3) The t-statistics in parentheses are as follows: ^*^ significant at the 10% level, ^**^ significant at the 5% level, and ^***^ significant at the 1% level

### Moderation effects of multilevel medical insurance systems

#### Social basic medical insurance

Air pollution leads to inequality in terms of medical costs, we analyse whether social basic medical insurance plays a role in improving inequality, and we add an interaction between social basic medical insurance and PM_2.5_. Table 7 presents the results regarding the moderating effect of social basic medical insurance. These results show that PM_2.5_ only affects out-of-pocket medical expenditures significantly (coefficient = 0.036, t-statistics = 2.23), and the coefficients of the interaction are not significant despite social basic medical insurance decreasing total (coefficient=-0.007, t-statistics =-0.02) and out-of-pocket (coefficient=-0.521, t-statistics =-1.13) outpatient expenditures and increasing reimbursements.

**Table 7:** The moderating effect of social basic medical insurance on the effect of PM_2.5_ on low-income residents

	Probit			OLS		
	(1)	(2)	(3)	(4)	(5)	(6)
PM_2.5_	-0.000	0.000	-0.002	0.016	0.014	0.036^**^
	(-0.06)	(0.07)	(-0.32)	(1.28)	(0.79)	(2.23)
interaction	0.085	0.075	0.117	-0.007	0.116	-0.521
	(0.73)	(0.60)	(0.71)	(-0.02)	(0.23)	(-1.13)
age	0.002	0.007	0.002	0.058^**^	0.048	0.055^**^
	(0.31)	(0.92)	(0.24)	(2.38)	(1.43)	(1.99)
age2	-0.000	-0.000	-0.000	-0.001^**^	-0.001	-0.001^**^
	(-1.15)	(-1.32)	(-0.94)	(-2.33)	(-1.52)	(-1.99)
gender	0.035	0.010	0.090^**^	0.295^**^	0.517^***^	0.045
	(0.94)	(0.22)	(1.96)	(2.17)	(2.74)	(0.31)
marriage	-0.068	-0.113	-0.062	-0.156	-0.347	-0.053
	(-0.98)	(-1.46)	(-0.75)	(-0.82)	(-1.64)	(-0.21)
edu	-0.010	0.006	-0.025	0.028	-0.023	0.026
	(-0.70)	(0.41)	(-1.56)	(0.44)	(-0.28)	(0.39)
fn	0.016^**^	0.020^**^	0.014^*^	0.028	0.054	0.009
	(2.16)	(2.12)	(1.69)	(1.26)	(1.57)	(0.27)
ur	0.002	-0.039	0.049	-0.193	-0.367^*^	-0.246
	(0.03)	(-0.52)	(0.75)	(-1.29)	(-1.94)	(-1.31)
hs	-0.268^***^	-0.270^***^	-0.227^***^	-0.280^***^	-0.225^***^	-0.309^***^
	(-13.11)	(-11.24)	(-9.16)	(-4.74)	(-2.71)	(-4.42)
pain	0.299^***^	0.311^***^	0.272^***^	0.158^***^	0.159^**^	0.106^**^
	(14.95)	(14.46)	(11.17)	(3.20)	(2.30)	(1.97)
smoke	-0.083^*^	-0.027	-0.116^**^	-0.172	-0.432^**^	-0.024
	(-1.65)	(-0.44)	(-2.16)	(-1.16)	(-2.23)	(-0.12)
drink	-0.182^***^	-0.154^***^	-0.139^**^	0.187	0.076	0.251
	(-3.63)	(-2.70)	(-2.46)	(1.14)	(0.38)	(1.18)
exercise	0.056	0.083^*^	0.092^*^	0.097	-0.019	-0.019
	(1.41)	(1.89)	(1.84)	(0.69)	(-0.11)	(-0.10)
inc	0.003	-0.005	0.018	0.014	0.019	0.004
	(0.34)	(-0.47)	(1.57)	(0.49)	(0.59)	(0.10)
GDP	-0.037	0.041	0.024	0.087	-0.305^**^	-0.136
	(-0.95)	(0.75)	(0.60)	(1.01)	(-1.99)	(-1.37)
med_ins	-0.005	0.004	-0.032	-0.069	-0.198^**^	-0.030
	(-0.17)	(0.09)	(-0.90)	(-1.01)	(-2.00)	(-0.47)
cons	-0.702	-2.243^***^	-1.330^**^	4.734^***^	10.124^***^	5.221^***^
	(-1.21)	(-2.74)	(-2.17)	(3.74)	(5.13)	(3.32)
sbmi	Yes	Yes	Yes	Yes	Yes	Yes
omi	Yes	Yes	Yes	Yes	Yes	Yes
Year effect	Yes	Yes	Yes	Yes	Yes	Yes
District fixed-effect	Yes	Yes	Yes	Yes	Yes	Yes
No. of observations	21,454	21,454	21,256	1342	896	816
R^2^	0.179	0.217	0.178	0.206	0.206	0.237

Notes: (1) Columns (1)-(3) present the incidence of total, reimbursed and out-of-pocket outpatient medical expenditures. Columns (4)-(6) present the amounts of total, reimbursed and out-of-pocket outpatient medical expenditures. (2) The figures in parentheses are clustered robust t-statistics at the level of cities. (3) The t-statistics in parentheses are as follows: ^*^ significant at the 10% level, ^**^ significant at the 5% level, and ^***^ significant at the 1% level

#### Supplementary medical insurance

As we mention in the introduction, with the improvement of the Chinese basic social medical security system, we have gradually recognized that basic and inclusive social basic medical insurance cannot meet the medical needs of residents and achieve a “fair and moderate” medical security mechanism, so we propose the concept of a “multilevel” medical security system. Under this policy background, we explore the supplementary medical insurance provided by corporations, which is a second-level medical security mechanism, to determine whether it improves the inequality in the medical costs of residents caused by PM_2.5_. We add an interaction between PM_2.5_ and supplementary medical insurance to the TPM, and the results are presented in Table 8.

For low-income residents, upon incorporating the interaction term of PM2.5 and supplementary medical insurance, their total medical expenses, reimbursement costs, and out-of-pocket expenses increase by 2.659% (t-statistics = 1.87), 3.225% (t-statistics = 2.02), and 4.915% (t-statistics = 2.72), respectively, for each 1% rise in PM2.5 concentration. In comparison to low-income residents without supplementary medical insurance, those with the insurance experience a reduction of 1.331% (t-statistics =-1.66) and 2.211% (t-statistics =-2.45) in the aforementioned increased reimbursement and out-of-pocket expenses, respectively, for each 1% increase in PM2.5 concentration. These results convincingly demonstrate the negative moderation effect of supplementary medical insurance on the increment of out-of-pocket expenses caused by PM2.5 for low-income residents.

**Table 8:** The moderating effect of supplementary medical insurance on the effect of PM_2.5_ on low-income residents

	Probit			OLS		
	(1)	(2)	(3)	(4)	(5)	(6)
PM_2.5_	1.093	1.369	1.232	2.659^*^	3.225^**^	4.915^***^
	(1.34)	(1.51)	(1.38)	(1.87)	(2.02)	(2.72)
interaction	-0.499	-0.630	-0.581	-1.111	-1.331^*^	-2.211^**^
	(-1.24)	(-1.40)	(-1.29)	(-1.54)	(-1.66)	(-2.45)
age	0.003	0.009	0.002	0.056^**^	0.046	0.057^**^
	(0.45)	(1.07)	(0.21)	(2.27)	(1.34)	(2.01)
age2	-0.000	-0.000	-0.000	-0.001^**^	-0.000	-0.001^**^
	(-1.28)	(-1.45)	(-0.90)	(-2.21)	(-1.42)	(-1.98)
gender	0.035	0.011	0.089^*^	0.288^**^	0.511^***^	0.037
	(0.94)	(0.24)	(1.93)	(2.12)	(2.75)	(0.25)
marriage	-0.069	-0.107	-0.070	-0.140	-0.330	-0.060
	(-1.00)	(-1.38)	(-0.86)	(-0.74)	(-1.53)	(-0.24)
edu	-0.007	0.010	-0.024	0.030	-0.020	0.036
	(-0.51)	(0.65)	(-1.49)	(0.48)	(-0.24)	(0.55)
fn	0.016^**^	0.020^**^	0.014^*^	0.028	0.053	0.011
	(2.19)	(2.10)	(1.69)	(1.24)	(1.57)	(0.32)
ur	-0.004	-0.047	0.050	-0.198	-0.394^**^	-0.249
	(-0.07)	(-0.61)	(0.77)	(-1.29)	(-2.03)	(-1.32)
hs	-0.268^***^	-0.271^***^	-0.226^***^	-0.281^***^	-0.217^**^	-0.313^***^
	(-12.87)	(-10.99)	(-9.12)	(-4.59)	(-2.54)	(-4.45)
pain	0.298^***^	0.310^***^	0.270^***^	0.153^***^	0.158^**^	0.099^*^
	(14.72)	(14.23)	(11.12)	(3.12)	(2.27)	(1.86)
smoke	-0.091^*^	-0.037	-0.119^**^	-0.163	-0.406^**^	-0.010
	(-1.83)	(-0.60)	(-2.21)	(-1.09)	(-2.10)	(-0.05)
drink	-0.182^***^	-0.155^***^	-0.134^**^	0.195	0.069	0.236
	(-3.59)	(-2.69)	(-2.37)	(1.19)	(0.35)	(1.11)
exercise	0.056	0.083^*^	0.088^*^	0.108	0.006	-0.031
	(1.40)	(1.88)	(1.80)	(0.76)	(0.04)	(-0.16)
inc	0.002	-0.006	0.017	0.014	0.019	0.004
	(0.21)	(-0.63)	(1.53)	(0.48)	(0.56)	(0.10)
GDP	-0.039	0.037	0.019	0.091	-0.302^**^	-0.107
	(-1.03)	(0.68)	(0.48)	(1.05)	(-2.00)	(-1.07)
med_ins	-0.008	0.002	-0.034	-0.087	-0.215^**^	-0.061
	(-0.24)	(0.05)	(-0.97)	(-1.24)	(-2.08)	(-0.92)
cons	-4.572	-7.069^**^	-5.694^*^	-4.133	-0.807	-11.239^*^
	(-1.52)	(-2.14)	(-1.78)	(-0.81)	(-0.14)	(-1.73)
sbmi	Yes	Yes	Yes	Yes	Yes	Yes
omi	Yes	Yes	Yes	Yes	Yes	Yes
Year effect	Yes	Yes	Yes	Yes	Yes	Yes
District fixed-effect	Yes	Yes	Yes	Yes	Yes	Yes
No. of observations	211,178	21,178	20,981	1328	885	809
R^2^	0.179	0.217	0.177	0.205	0.205	0.234

Notes: (1) Columns (1)-(3) present the incidence of total, reimbursed and out-of-pocket outpatient medical expenditures. Columns (4)-(6) present the amounts of total, reimbursed and out-of-pocket outpatient medical expenditures. (2) The figures in parentheses are clustered robust t-statistics at the level of cities. (3) The t-statistics in parentheses are as follows: ^*^ significant at the 10% level, ^**^ significant at the 5% level, and ^***^ significant at the 1% level

## Discussion

According to the results of the basic regression and robustness tests, we know that PM_2.5_ has a positive effect on residents’ medical expenditures and that their total, claimed and out-of-pocket outpatient expenditures increase with an increase in PM_2.5_, which is consistent with the results of other studies using provincial-level data or old micro data [17, 18, 28]. Moreover, the coefficients of PM_2.5_ indicate that air pollution has a greater influence on reimbursed expenses than on other expenses; this phenomenon is related to the gradual improvement of the basic medical insurance mechanisms in China and the increasing awareness of health insurance among its residents.

Generally, air pollution increases residents’ medical expenditures by affecting their health, and it has been proven that air pollution leads to unfair health effects across people of different socioeconomic status [19–23]. The results show that PM_2.5_ has no significant effects on the medical costs of high-income residents, while it significantly affects the total and out-of-pocket medical expenditures of low-income residents. Compared with the basic results, there are two differences in the results regarding low-income residents. First, although air pollution increases the total and out-of-pocket medical expenditures of the low-income residents, this increase in reimbursement is no longer significant, which suggests that the medical burden of low-income residents is greater than that of high-income residents. Second, the influence of PM_2.5_ concentration on the low-income residents is greater than that on the whole sample of residents, which indicates that air pollution has a more serious effect on low-income residents than on high-income residents. Importantly, air pollution results in inequality in terms of medical costs. These findings align with the results indicating that air pollution contributes to health inequality among residents, consequently leading to disparities in medical expenditures [19–23].

Previous studies discuss the fact that air pollution intensifies unfair health status among residents, especially using Chinese data or in the context of the Chinese medical security system, and propose that medical services should be equalized or the establishment of a universal medical security system should be accelerated [5, 16, 23, 32, 33]. However, there is scarce research investigating the role played by basic medical insurance in the process. Our results suggest that social basic medical insurance intended to guarantee residents’ basic medical needs does not effectively improve the inequality in health benefits caused by the air pollution faced by low-income residents. On the contrary, it is the supplementary medical insurance provided by corporations, serving as the second tier of the multilevel medical insurance system, that effectively mitigates the inequality in medical costs caused by air pollution.

We discuss the following two reasons for the result. On the one hand, the coverage of social basic medical insurance reaches 96%, which is nearly full coverage. However, most high-income residents are covered by urban employee basic medical insurance (UEBMI) with high financing levels and diverse treatments, while low-income residents are generally covered by low-cost URBMI and NRCMS with limited treatments [34]. As a result, social basic medical insurance does not mitigate the unfair health costs caused by pollution that low-income residents face. There is a strong pro-rich inequality in both the probability and the frequency of use for health services among the elderly in China, and medical insurance is not enough to address this inequity [35]. On the other hand, supplementary medical insurance is commercial health insurance in which employers and employees enroll voluntarily with the encouragement of local governments’ policies. The government and corporations pay most of the premiums, and residents pay small premiums according to their salaries; thus, this system is a type of welfare intended to provide medical security to residents and improve the unfair health costs of low-income residents in a targeted way.

## Conclusion

The study concludes that air pollution augments residents’ outpatient expenditures and exhibits no significant impact on the incidence of outpatient costs. However, the influence of air pollution is more pronounced among low-income residents compared to their high-income counterparts, indicating that air pollution contributes to the inequity in medical costs. Furthermore, supplementary medical insurance mitigates the disparity in medical costs resulting from air pollution for low-income employees.

## Data Availability

All the data were available from Social Sciences Survey Center of Sun Yat-sen University for China Labour Force Dynamics Survey, http://css.sysu.edu.cn. And we would like to inform our data code to reviewers.
